# Local dynamics of a white syndrome outbreak and changes in the microbial community associated with colonies of the scleractinian brain coral *Pseudodiploria strigosa*

**DOI:** 10.7717/peerj.10695

**Published:** 2021-02-02

**Authors:** Patricia E. Thome, Jacqueline Rivera-Ortega, Jenny C. Rodríguez-Villalobos, Daniel Cerqueda-García, Edgar O. Guzmán-Urieta, José Q. García-Maldonado, Natalia Carabantes, Eric Jordán-Dahlgren

**Affiliations:** 1 Instituto de Ciencias Del Mar y Limnología, Unidad Académica de Sistemas Arrecifales, Universidad Nacional Autónoma de México, Puerto Morelos, Quintana Roo, Mexico; 2 Departamento de Ciencias Marinas y Costeras, Universidad Autónoma de Baja California Sur, La Paz, Baja California Sur, Mexico; 3 Ecosistemas y Conservación, ProAzul Terrestre A.C., La Paz, Baja California Sur, Mexico; 4 Centro de Investigación y de Estudios Avanzados del IPN, Unidad Mérida, Departamento de Recursos del Mar, Instituto Politécnico Nacional, Mérida, Yucatán, Mexico; 5 CONACyT, Centro de Investigación y de Estudios Avanzados del IPN, Unidad Mérida, Mérida, Yucatán, Mexico

**Keywords:** Coral disease, White syndrome, Survival analysis, Microbiome, 16S rRNA, White plague II, SCTLD, Histopathology

## Abstract

Reef corals in the Mexican Reef System have been severely affected by the emergence of a white syndrome that resembles both White Plague II and SCTLD descriptions. Meandroid scleractinian coral species are among the most severely affected. To gain insight into this affliction we conducted a broad study in the brain coral *Pseudodiploria strigosa* at a rear reef site in the NE Mexican Caribbean. We describe macro and microscopical signals of the disease, characterize the outbreak dynamics, the tissue histopathology, explore immunological responses in the individuals, and compare microbial assemblages associated with the surface mucus layer of healthy and unhealthy colonies. At the study site, the white syndrome outbreak on *P. strigosa* showed a high incidence rate in summer-fall and a low one in winter, as well as low survival expectation of diseased colonies at the end of the study. After 306 days of observation, out of 96 tracked colonies, eight remained apparently healthy and seven were diseased. No effective resistance to colony disease progression was observed once white syndrome signs developed. Tissue loss rate during the study varied among colonies (mean = 10.8 cm^2^, s.d. = 7.8 cm^2^) suggesting a complex relation between causal agents and colony resistance. The deterioration of tissues was evidenced from the basal to the surface body wall of polyps (up to 66% hypertrophy and liquefactive necrosis in unhealthy colonies), implying that microscopic alterations begin before macroscopic signals develop, suggesting this may be a systemic disease. We measured high levels of phenoloxidase (two orders of magnitude higher PO activity than *P. strigosa* affected by BBD) and antibacterial activity without significant reduction in unhealthy samples from the mucus layer, indicative of an enhanced immunological response. Results showed that opportunistic bacteria dominated damaged colonies, where six genera of the Bacteroidia class were found with significant changes in unhealthy colonies after DeSeq2 analysis. Nevertheless, histological observations did not support infection of the tissues. The opportunistic overload seems to be contained within the mucus layer but may be associated with the mortality of tissues in a yet unclear way. Future research should focus on experimental infections, the tracking of natural infections, and the immunocompetence of corals in the face of environmental pressures due to local, regional, and global impacts. If environmental deterioration is the primary cause of the continuing emergence and re-emergence of lethal coral diseases, as has been proposed by many authors, the only true option to effectively help preserve the coral reef biodiversity and services, is to restore the environmental quality of reef waters at the local scale and reduce greenhouse gases at the global scale.

## Introduction

One of the major threats to the persistence of coral reefs are coral diseases. The deterioration of the reef environment mainly due to anthropogenic causes, has resulted in coral disease outbreaks being more frequent and more damaging (Weil, 2004; Van Woesik & Randall, 2017). Climate change can further accelerate the occurrence, prevalence, and incidence of coral diseases (Bruno et al., 2007; Harvell et al., 2007), causing many reefs to experience a significant reduction in the cover of reef-building species (De Bakker et al., 2016) and the loss of critical functional processes (Estrada-Saldívar et al., 2019). Since the summer of 2018, reef corals in the Mexican Caribbean have been affected by the emergence of a white syndrome (WS) that partially resembles both, white plague type II (WP-II) (Richardson et al., 1998a; Richardson et al., 1998b), and a stony coral tissue loss disease (SCTLD) (Florida Keys National Marine Sanctuary, 2018; Meyer et al., 2019). Both diseases affect many species and their lethality is high. The overall disease spatial spread patterns resemble the epizootics reported for a WP by Croquer, Weil & Zubillaga (2005) in Venezuelan reefs, as well as those in Florida reefs (Precht et al., 2016; Walton, Hayes & Gilliam, 2018). Although the etiology for these diseases is unknown, it is assumed that recent WS like outbreaks in the US Virgin Islands and Dominican Republic (AGRRA, 2019), as well as in the Mexican Caribbean (Alvarez-Filip et al., 2019) correspond to the SCTLD. However, the characterization of causative agents for white plagues (Pantos et al., 2003; Sunagawa et al., 2009) or the SCTLD (Aeby et al., 2019) seem so far elusive, giving a plausibility of multiple causes that can even change in time (Work, Russell & Aeby, 2012). Therefore, in this study, we have chosen to use the generic term of “white syndrome” to avoid adding more ambiguity to the study of coral diseases.

Corals, as holobionts, harbor a microbial community with functional roles that promote the survival and health of hosts (Rosenberg et al., 2007; Gates & Ainsworth, 2011). However, disease outbreaks can change the structure of this microbial community drastically (Sweet & Bulling, 2017; McDevitt-Irwin et al., 2017), and may involve a consortium of microbes, making the characterization of particular coral diseases challenging (Pantos et al., 2003; Bythell, Pantos & Richardson, 2004; Aeby et al., 2019). Environmental stresses hamper key functional coral processes and may lead to immunosuppressed conditions (Harvell et al., 2007), opening the possibility to similar disease’s signs resulting from different opportunistic pathogens (Lesser, Bythell & Gates, 2007). Moreover, distinguishing among white tissue loss diseases, such as white plagues, white syndromes, or SCTLD, merits the identification of the etiologic agent(s) or pathogen(s) and the characterization of their pathogenesis.

Some coral diseases are associated with pathogenic microorganisms. For example, the WP-II was related to three different pathogens, *Shingomonas* sp. (Richardson et al., 1998a), *Thalassomonas loyana* (Thompson et al., 2006), and *Aurantimonas corallicida* (Denner et al., 2003). However, later studies did not detect these purported pathogens in corals affected by the disease (Pantos et al., 2003; Sunagawa et al., 2009). Coral diseases can result from opportunistic microorganisms that proliferate under conditions of environmental pressure leading to coral stress (Lesser, Bythell & Gates, 2007; Maher et al., 2019), or to microbial imbalance that breaks the homeostasis of the coral holobiont (Harvell et al., 2007; Lesser, Bythell & Gates, 2007; Glasl, Herndl & Frade, 2016; Egan & Gardiner, 2016). Considering that generic antibiotics seem to halt the progression of lesions in diseased colonies in Florida (Aeby et al., 2019), this WS outbreak could be associated with the presence of biotic agents.

Corals employ diverse immune strategies to protect themselves. The synthesis of melanin is activated by pathways associated with invertebrate immune responses and is mainly used to entrap potential pathogens (reviewed in Palmer & Traylor-Knowles, 2018). In addition, phenoloxidase is an enzyme involved in the synthesis of melanin (Palmer, Mydlarz & Willis, 2008; Mydlarz et al., 2009) whose activity can also be detected in the Surface Mucus Layer (SML) (Rivera-Ortega & Thomé, 2018). This enzyme produces toxic intermediates, such as cytokines and reactive oxygen species, and its activity increases in corals challenged by pathogens, bleaching, and injuries (Palmer, Mydlarz & Willis, 2008; Mydlarz et al., 2009; Palmer, Bythell & Willis, 2010; Van der Water et al., 2018). Further, the SML covering corals acts as a protector, containing antibacterial properties that inhibit the growth of pathogenic microorganisms (Ritchie, 2006). The antibacterial activity of the SML is thus implicated in disease resistance (Ritchie, 2006; Gochfeld & Aeby, 2008; Shnit-Orland & Kushmaro, 2009).

Understanding the etiology of a disease requires considering not only the development of the disease at the population level, but at an individual scale (Bythell, Pantos & Richardson, 2004; Work & Metemeyer, 2014). Given the complexity of factors that may be involved in coral diseases and the urgency to reach a better understanding of the recent white syndrome epizootic, in this study we aimed to disentangle this disease through a broad approach. In addition to describing the outbreak effect on a natural setting, we studied the macroscopic and temporal evolution of WS lesions in selected colonies, together with histological observations. These macro and microanalysis are also useful for the case definition of diseases and can help to identify potential etiologic agents associated with the disease (Work & Metemeyer, 2014; Rodríguez-Villalobos et al., 2014). The immunological responses of damaged colonies were studied through several parameters. We also looked at the composition of the bacterial community at the SML of healthy and unhealthy colonies, to help identify potential pathogens or perhaps a disruption of the associated microbial community (Shnit-Orland & Kushmaro, 2009; Glasl, Herndl & Frade, 2016; Egan & Gardiner, 2016; Sweet & Bulling, 2017).

## Materials & Methods

### WS outbreak follow-up

*Pseudodiploria strigosa* is an abundant reef building coral species at our study site, located near Puerto Morelos in the NE Mexican Caribbean (20°53′04″N, 86°50′50″W). The study site is characterized by a dense coral assemblage with more than 14 scleractinian species spatially distributed so that few of them are in direct contact with each other. We selected *P. strigosa* colonies given their characteristic high abundance and also because it seemed to be one of the most severely affected species (Alvarez-Filip et al., 2019). Ninety-six colonies of *P. strigosa* were monitored during a WS outbreak in this reef site. Each colony condition was followed through direct observation and photographic records in surveys carried out at non regular intervals (dates of surveys are given in Fig. S1), until most of the colonies showed signs of the WS disease or died (306 days in total, initiating on August 22nd, 2018). Initial colony’s photographs included a 5 cm scale to allow for colony size estimations as colony projected surface area, using the free program ImageJ. The progression of lesions was assessed by measuring tissue loss on consecutive images.

Initial WS prevalence (number of diseased colonies/total number of colonies × 100), incidence rates (IR), and survival probability were estimated for *P. strigosa* at the study site. The incidence rate describes how quickly an event (developing signs or dying) occurs in the sampled population at risk; the index calculation accepts colonies leaving or entering the study by incorporating time directly into the denominator of the equation. In our study, some colonies died before the study ended, others were lost for a given survey, and some others entered the study later, therefore each colony has its own tracking time. The average IR for the whole study time for both sets of colonies at risk (becoming diseased or dying if already diseased) were calculated as the total number of new cases divided by the total colony-time observed (Olsen et al., 2010). A more detailed description of the WS dynamics was accomplished by estimating the IR at each survey period for both sets at risk; these incident rates for each survey were calculated as the number of new cases per time interval between surveys, divided by the total colony-days observed during the interval. The number of colony-days were adjusted for mid interval times whenever a colony developed signs or died, since it is not known when the event occurred exactly within each interval (actuarial method; Benichou & Palta, 2005) and thus, colony time contribution is half of the corresponding interval. The data from the first survey were not considered in the analyses, unless the colony condition was apparently healthy.

To complete the description of the WS dynamics at the study site, survival probability of diseased colonies since the onset of disease (emergence of WS signs) was estimated with a non-parametric maximum likelihood estimator for interval censored data (R survival package; Therneau, 2015) and expressed as Kaplan–Meier survival curves. Comparisons of survival probability as a function of colony size were assessed by means of log-rank non-parametric tests for interval data (R interval package) that assumes disease and mortality onset times as the start and endpoint of the interval for each colony at risk of dying (Fay & Shaw, 2010), and the step curve reflects the fact that it is not known when along the interval the death event occurred. We also described gross lesions for the surveyed colonies through direct observations and photographic detailed analysis following the protocol by Work & Aeby (2006).

### Coral tissue and mucus sampling

Coral samples were collected from 12 colonies of *P. strigosa* between September and October 2018, under permit number PPF/DGOPA-033/19 from CONAPESCA. The sampled colonies were scattered throughout the same coral reef site and their condition is shown in Table S1. We compromised for a low number of colonies to be sampled, to minimize the impact of sampling in the face of the unknown outbreak extent. Six out of the 12 colonies had no visible signs of white syndrome (named healthy), and six colonies showed visible signs of the white syndrome (named unhealthy). Two core samples (2 cm diam. to a depth of 2–3 cm into the skeleton of colonies) were collected from unhealthy colonies, one close to the edge of the lesion (UCL), and another sample 15 cm away from the lesion over healthy-looking tissue (UAL) (Fig. S2), to make a total of 18 samples (six healthy, 12 unhealthy). Also, one liter of seawater next to one healthy and one unhealthy colony was collected as background reference. After collection, each sample core was individually stored in a hermetic container and transferred to the lab in a cooler. At the lab, mucus (ca. 150 µl per core) from each sample was collected with a sterile micropipette after an air exposure (20 min) in a sterilized chamber. We processed 10 µL of mucus for DNA extraction. The rest of the mucus was either used fresh for antibacterial assays or refrigerated (7 °C) until immunological assays (2–3 days). After mucus collection, the cores were individually stored with 10% buffered formalin in filtered seawater and mailed to the Histology Lab at UABC for processing.

### Histological observations

Fixed samples were rinsed in freshwater and preserved in 70% ethanol until decalcification with HCL 10% buffered solution (0.7 g EDTA, 0.14 g sodium tartrate, 0.008 g potassium sodium tetrahydrate). Tissues were then embedded in paraffin and sectioned (5 mm thick). Samples were stained with Harris’s hematoxylin and eosin routine procedure (Humason, 1967) for histopathological analysis. Microscopic morphological alterations were assessed in comparison to normal tissues from healthy and unhealthy colonies. Observations were done at different magnifications (x4, x10, x40) using a Leica DM500 light microscope (Leica, Germany) equipped with a Leica ICC50 HD camera. Images were processed using both LAS imaging software (Leica, Germany) and Adobe R Photoshop CC 2015.5®. Tissues were divided for histological interpretation into surface body wall (SBW) comprising the coenenchyme and polyp, and the basal body wall (BBW) including mesenterial filaments. We recorded the observed changes per colony as atrophy, hypertrophy, sloughing of epithelia from mesoglea, zooxanthellae depletion, or malformation. The presence of ciliates, bacterial aggregates, and suspect endolithic algae or fungi were also recorded on the datasheets to relate gross and microscopic pathology findings. Results are shown as the percentage of occurrences, considering six colonies as the total sample size for each condition.

### DNA extraction for bacterial analysis

Mucus samples (10 µL) were centrifuged and then transferred to lysis tubes for genomic DNA extraction, adding extraction buffer. Seawater samples were filtered through 2.5 µm filters (Whatman™) followed by the collection of microbes by filtering with Durapore® membranes (0.45 µm and 0.22 µm). DNA from the centrifuged mucus and the microbes collected on the 0.45 and 0.22 µm filters, was extracted with DNeasy® PowerBiofilm Kit. DNA integrity was evaluated by electrophoresis in 1% agarose gels. DNA concentration and quality were assessed and only samples with adequate parameters (DNA concentration ≥ 100 ng, A_260∕280_ of 1.6 to 2.0) were processed further.

### 16S rRNA Illumina MiSeq libraries preparation

PCR amplification of the hypervariable V3 and V4 regions of the 16S rRNA gene was performed using the primers and the conditions suggested by Klindworth et al. (2013). We used primers 341F 5′-CCTACGGGNGGCWGCAG-3′ and 805R 5′-GACTACHVGGGTATCTAATCC-3′ with Illumina overhanging adapters attached. Indexed PCR products were purified and quantified with a Qubit® 3.0 Fluorometer (Life Technologies, USA). Amplicon size was verified by capillary electrophoresis and the sequencing was carried out in CINVESTAV-Mérida using an Illumina-MiSeq platform (Illumina, USA), with the MiSeq reagent kit V3 (2 × 300), following the manufacturer’s recommendations.

### Analysis of amplicon libraries

Paired end 2 × 250 reads were processed with the QIIME 2 pipeline (https://docs.qiime2.org/, see Supplementary Reference). After manual inspection, both forward and reverse reads were trimmed in position 40 from the 5′ end and truncated in position 250 from the 3′ end, to filter out low quality positions (bases with Q-score <20). DADA2 plugin (Callahan et al., 2016) was used for denoising, error correction, and removing of chimeras, to finally resolve ASVs (Amplicon Sequences Variants, unique sequences indicative of distinct taxa). The taxonomic assignment of the representative sequences of the ASVs was done with the classify-consensus-vsearch plugin (Rognes et al., 2016), using SILVA 132 database as reference. Representative sequences were aligned and masked with MAFFT (Katoh & Standley, 2013), a phylogenetic tree was built with FasterTree 2 (Price, Paramvir & Arkin, 2010). The feature table was filtered for chloroplast and mitochondrial ASVs and imported into the R environment. The feature table was rarefied to 16,600 counts per sample, and the statistical analysis performed with phyloseq (McMurdie & Holmes, 2013), Vegan (Oksanen et al., 2009), and ggplot2 v3.1.0 (Wickham, 2016) libraries. Alpha diversity indexes (Shannon, Simpson, Chao1, and observed ASVs) were calculated. An analysis to identify the ASVs with differential abundance among conditions was performed using the DESeq2 analysis with internal normalization (Love, Huber & Anders, 2014). A phylogenetic analysis was applied to the most abundant ASVs of unhealthy samples. The ASVs were searched for their closest homologs with the blastn program in the RefSeq NCBI database. The ASVs sequences and their best hits were aligned with MUSCLE (Edgar, 2004). The alignment was trimmed, and a phylogenetic tree generated with PhyML with the GTR+I substitution model, supporting branches with the aLRT method (Guindon et al., 2010).

### Mucus treatment and immunological assessment

The mucus was processed in two different treatments, the Surface Mucus Layer (SML or mucus without bacteria) and the Mucus Complex (MC or the mucus with its microbiome, without treatment). For the SML treatment, the mucus was sterilized for 20 min under UV light (254 nm) and its effect corroborated by incubating an irradiated sample on marine agar (Sobel Marine Agar, Difco) at 27 °C overnight and checking for the absence of bacterial growth. For antibacterial activity assessments, we employed a swarming assay combined with a double agar overlay assay, as previously described (Rivera-Ortega & Thomé, 2018), challenging the SML and MC samples against two potential coral pathogens, *Aurantimonas* sp. (strain BAA-667, ATCC) and *Serratia marcescens* (strain BAA-632, ATCC) (Patterson et al., 2001; Denner et al., 2003). The presence of an inhibition zone was identified under a stereoscopic microscope.

We looked for the presence of melanin in tissues, evidenced as golden deposits in histological slides (Palmer, Mydlarz & Willis, 2008), as well as by the activity of phenoloxidase (PO) in mucus samples. For the quantification of the specific activity of PO in mucus, we followed a protocol described in Mydlarz & Palmer (2011), using 10 µL of mucus from each sample, with three technical replicates. Results are presented as a change in absorbance A_490_ mg protein^−1^ min^−1^. Total protein in mucus samples was measured by the Bradford method against a standard curve prepared with BSA.

### Statistical analyses

Survival probabilities of diseased colonies at risk of dying were estimated by means of a non-parametric maximum likelihood estimator (NPML) for interval censored data, to take into account the unequal interval monitoring (Therneau, 2015). All statistical analyses were performed in R v.3.6.1. For the antibacterial assays, we applied the exact Fisher test to compare differences among treatments for each tested bacterium. This test is adequate for contingency table analyses with categorical data and small sample sizes. To analyze the statistical differences of the PO activity in the mucus among treatments, we applied a Kruskal–Wallis test since the data was not normally distributed. In all tests, statistical significance was considered at *p* < 0.05.

## Results

### White syndrome (WS) outbreak at the study site

**Figure 1:**
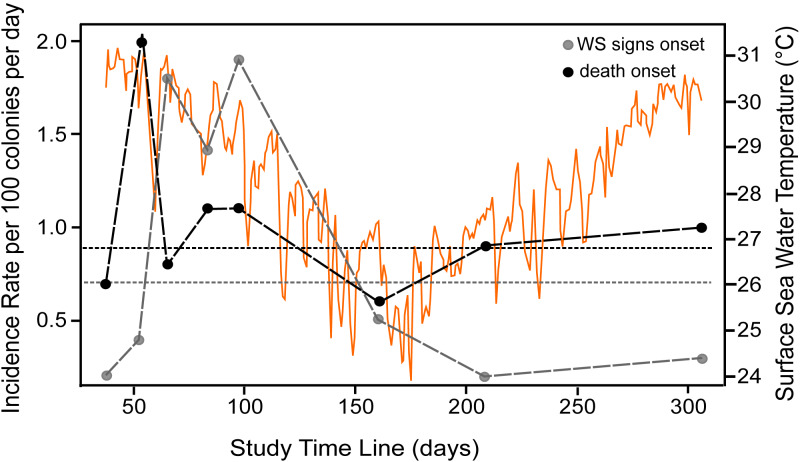
Incidence rate of disease signs and death per survey. Incident rate as calculated for colonies at risk of developing WS signs (grey dots) and of diseased colonies of dying (black dots) at the study reef site. Average incidence rate (horizontal short-dotted lines) of developing WS signs (grey) and of diseased colonies of dying (black). The *X*-axis indicates the time span of the study in days, starting from August 22nd, 2018. The dots indicate the number of survey days and the length of each interval between consecutive surveys. Orange graph shows daily sea surface temperatures during the study at the reef site (data from SAMMO, 2020). See Fig. S1 for survey’s dates.

At the beginning of the study (August 2018), WS prevalence in *P. strigosa* colonies was relatively high (5.2%; CI [2.2%–112.9%]). At the end of the study (306 days of observation), out of 96 tracked colonies, eight remained apparently healthy and seven were diseased. The WS outbreak on the monitored colonies showed a progressive pattern of cumulative appearance of signs and colony mortality (Fig. S1). Average incidence rates (IR) for the whole study of healthy colonies at risk of developing WS signs was 0.007/colony-days (for 100 healthy colonies at risk, 0.7 colonies will develop WS signs every day). Incidence risk per survey (IRs) showed two stages: (1) a sudden increase and maintenance of a relatively high IRs in the summer-fall of 2018, when apparently the most susceptible colonies at risk developed WS signs, with an average of 0.012/colonies per day; and (2) a relatively low IRs phase starting in winter (0.005/colonies per day), gradually affecting some of the remaining susceptible colonies of the population at risk to develop WS signs (Fig. 1). For diseased colonies at risk of dying, the average IR was 0.009/colony-days. The isolated peak early in the study (Fig. 1, IR for diseased colonies at risk of dying) is associated with a relatively large number of dead colonies (*n* = 6) for the observation period (298 colony-days), doubling the average IRs which, except for the spike, correspond with the average IR value. The spike does not seem associated with specific environmental conditions, but with the occurrence of multiple WS lesions in three of the six dead colonies, which rapidly succumbed to the disease. There was a significant correlation (Spearman rho = 0.89, *p* = 0.003) between the number of new dead colonies and time at risk in days per survey, supporting the constancy of the risk of dying once a colony developed WS signs.

**Figure 2:**
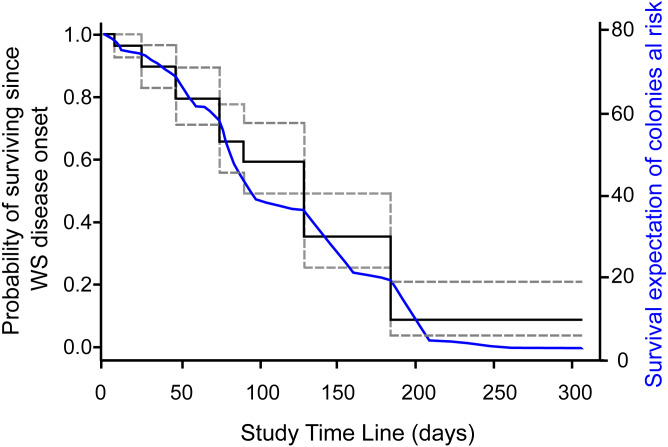
Survival curve of *Pseudodiploria strigosa* colonies at the study site. Fitted NPMLE values into a Kaplan–Meier survival curve for interval censored data for each survey. Solid black line indicates average survival for the interval among surveys and dotted lines indicate the width of the 95% survival confidence interval during survey intervals. The solid blue line shows the fitted expectation of surviving colonies at risk of dying.

Survival probabilities of diseased colonies at risk of dying were consistent with IR of colonies at risk of dying per survey estimates, where the probability of colony survival drops more or less constantly from the first survey to 0.09 (95% CI [.04–.21]) by the end of the study, as shown in the overall Kaplan–Meier survival curves (Fig. 2). To assess if colony size was related to colony survival probability, we arbitrarily divided the colonies at risk of dying into groups by quantiles (0.2, 0.4, 0.6, 0.8), as to categorize size values into small, medium, large, and very large colony sizes, as the log rank test for interval surveys does not perform well with continuous data. Contrary to expectations, the test found no significant differences (Chi Square = 3.73, *p* = 0.29) among the survival curves of the different groups, which in general showed the same strong decreasing trend indicated in Fig. 2. Tissue loss speed due to WS varied among colonies, occurring very fast in two cases (41 and 72 cm^2^ per day), but much slower in the remaining colonies with an average of 10.8 ± 7.8 cm^2^ per day.

**Figure 3:**
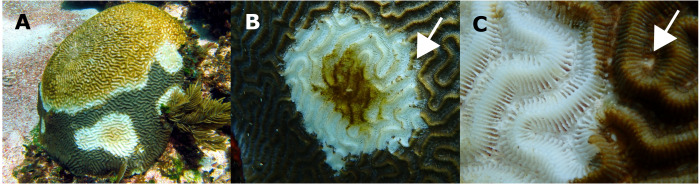
*Pseudodiploria strigosa* colonies with signs of a White Syndrome in our study area. (A) Multifocal to coalescing. (B) The white arrow shows residuals of normally pigmented tissue into the white intact bare skeleton. (C) The white arrow shows a discolored polyp mouth. Photo credits: (A) Eric Jordán-Dahlgren (B, C) Jacqueline Rivera-Ortega.

Macroscopic signs presented high morphological variations (Table 1). According to Work & Aeby (2006) categorization, colonies showed focal, multifocal, and coalescing lesions that were subacute to acute, usually starting as a small spot anywhere on a colony (Table 1). Some colonies developed several lesions, but in all cases, the exposed intact bare skeleton was rapidly covered by turf, with remnant tissue normally pigmented (Fig. 3).

**Table 1:** Description of lesions for *Pseudodiploria strigosa* colonies affected by white syndrome in our study area.

Categories	Description of lesions
Distribution	Focal, multifocal, coalescing
Size	Small to large
Location	Basal, medial, apical
Shape	Circular, oblong, irregular
Edges	Distinct
Lesion	Sharp line of tissue loss exposing the skeleton
Color	Pale to colorless (bleached margins?)
Margins	Smooth
Extent	Mild to severe
Time	Subacute to acute
Structures Affected	Polyp, coenosarc

### Histological observations

**Figure 4:**
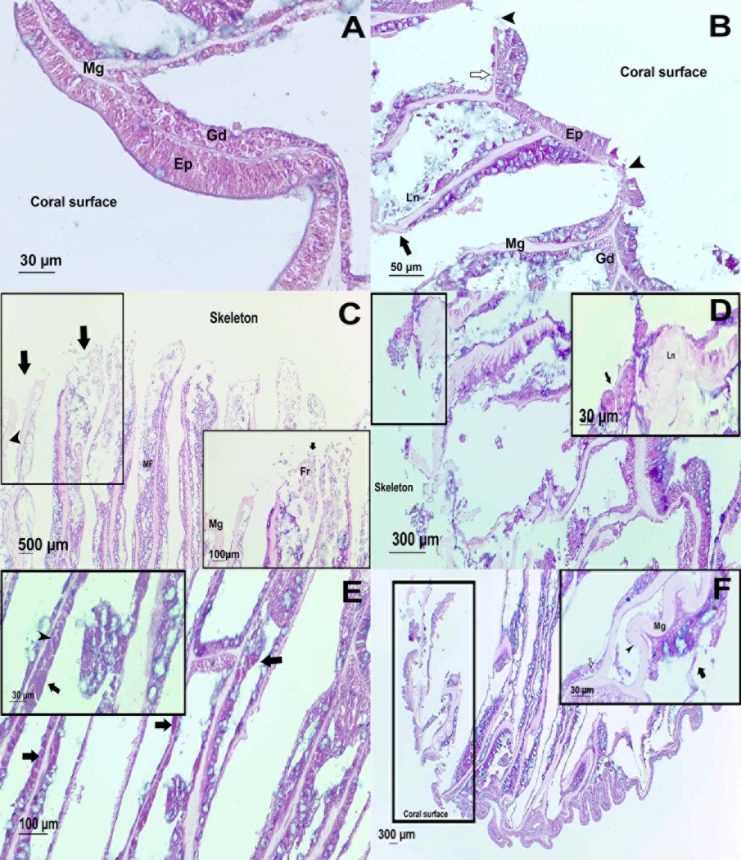
Histological observations on healthy and affected *Pseudodiploria strigosa* colonies. Surface body wall (SBW) of a (A) healthy and (B) WS affected colonies. Note in the affected sample (black arrowhead) the fragmentation of the epidermis (Ep), the thinning (white arrow) gastrodermis (Gd), and the liquefactive necrosis (Ln) that left a nude mesoglea (Mg). Basal body wall (BBW) of affected colonies (C). Fragmentation (black arrow) of calicodermis, gastrodermis, and mesoglea (black arrowhead) in the mesenterial filaments (MF) in an UCL sample. Inset: close up of the fragmented (Fr) tissues. (D) General atrophy of the BBW in an UCL sample. Inset: ciliates (black arrow) in near of liquefactive necrosis (LN). (E) Note in the BBW the necrosis in the Gd (black arrow) and some remaining zooxanthellae (black arrowhead) in an UCL sample. (F) Alterations of the SBW of a healthy colony (no macroscopic manifestations of disease). Fragmentation and ablation of tissue from Mg. Inset shows a close up of the mesentery presenting fragmentation (black arrow), remaining zooxanthellae (white arrow) in the fragmented Gd and nude Mg (black arrowhead).

The surface body wall (SBW) in healthy colonies consisted of an epithelium of pseudostratified columnar cells, clearly separated from the gastrodermis by mesoglea (Fig. 4). The gastrodermis comprised a simple layer of cuboidal cells hosting symbionts, with distinct nuclei and pyrenoids. Tissues from UAL and UCL samples, manifested alterations associated with tissue damage such as hypertrophy (66%, *n* = 4 in UCL, 17%, *n* = 1 in UAL) and liquefactive necrosis (33%, *n* = 2 in UCL, 66%, *n* = 4 in UAL). These changes were evident within polyps from the basal body wall (BBW, Figs. 4B and 4D) (calicodermis and gastrodermis) to the SBW (Fig. 4A), that in general, presented minor signals of damage. In some cases, in samples from healthy (33%, *n* = 2) and UAL colonies (50%, *n* = 3), we observed tissue fragmentation from the SBW of the polyps, but with the epidermis of the SBW in good condition (Figs. 4C and 4F). Ablation of tissue from mesoglea and hypertrophy of the epithelia was frequently observed in samples from affected (UCL, 85%, *n* = 5; UAL, 50%, *n* = 3) and unaffected colonies (H, 33%, *n* = 2) (Figs. 4D, 4E and 4F). We did not find evidence of bacteria or crystalline inclusion bodies near the affected tissue; however, in the surrounding areas of the deteriorated calicodermis, we usually found microalgae inclusions (UCL, 50%, *n* = 3; UAL, 17%, *n* = 1), cyanobacterial mats (17%, *n* = 1), and ciliates (UCL, 17%, *n* = 1; UAL, 17%, *n* = 1) in association with necrosis of the tissues (Fig. S3).

### Bacterial community analysis

We successfully sequenced three healthy, three UCL, and two UAL samples from the original 18 samples collected (6 of each). We obtained a total of 261,176 clean reads which were normalized to 16,600 per sample (see species richness curves, Fig. S4) and finally grouped in 1,167 ASVs (Table S2). Proteobacteria was the dominant Phylum in all samples, except in the mucus layer of UCL samples, where Bacteroidetes and Firmicutes exhibited high relative abundances (Fig. S5). At lower taxonomic level, Gamma- and Alpha-proteobacteria were dominant classes in healthy and UAL samples (Fig. S5) while Bacteroidia, Gamma-proteobacteria, Clostridia, and Bacilli were well represented for the UCL samples. The number of shared ASVs between healthy and UCL samples, and those between UAL and UCL samples, was the same (Fig. S6). Alpha diversity varied in all sampled colonies without a clear pattern (Table S3).

**Figure 5:**
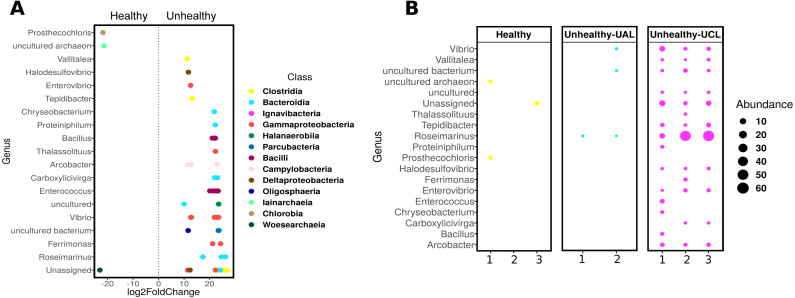
Differential abundance of ASVs in *Pseudosiploria strigosa* colonies with or without signs of White Syndrome. (A) ASVs with significant fold-change (calculated as log2) at genus level, detected in the bacterial community of healthy and unhealthy (close to lesions or UCL) colonies. Data obtained from DESeq2 analysis. The color of the circles depicts the taxonomic Class level. (B) Relative abundance of the genera identified by DESeq2 analysis, for every colony sampled.

Even though there were a low number of samples, it was possible to apply a differential abundance analysis (DESeq2) to ASVs from healthy and UCL colonies (Fig. 5). This analysis showed the enrichment of three genera in healthy colonies, but 17 genera in unhealthy colonies (Fig. 5A). In healthy colonies, Chlorobia, Iainarchaeia, and Woesearchaeia were significantly different in comparison with the unhealthy colonies near the lesions, where six genera of the Bacteroidia class were found with specific changes (Fig. 5A). Moreover, other abundant genera such as *Vibrio*, *Enterococcus*, and *Arcobacter* were observed in unhealthy colonies (Fig. 5B). The classes Bacteroidia (seven genera), Gamma-proteobacteria (five genera), and Clostridia (three genera) had the most genera that changed in unhealthy colonies. The differential abundance among bacteria from the three colony conditions consisted of 53 ASVs (Fig. 5B). Within the class Bacteroidia, the genus *Roseimarinus* was particularly enriched. This genus had a relative abundance of 39.27 ± 23.37% on average for UCL samples. Seven different ASVs in the genus *Roseimarinus* were detected in unhealthy colonies (Table S2). To gain insight into the phylogenetic relationship of *Roseimarinus* ASVs we did a phylogenetic analysis, including also other abundant ASVs identified in unhealthy colonies (Fig. S7). The most abundant *Roseimarinus* ASV (tag = ebecc93ba) was clustered with *Marinilabilia nitratireducens* at 89.3% similarity, the closest relative known; also, for the ASV assigned with JTB215 Clostridia Firmicutes (tag = 5bb6936d9b4) the closest relative known is *Peptoclostridium litorale* with 89.5% similarity; while all the other ASVs were clustered with the same genus as assigned by the SILVA database. Finally, we show the relative abundance of the orders Rhodobacterales and Rhizobiales in sampled colonies, given their probable importance for white syndromes (Table S4), although our results indicate a reduction in the relative abundance of members in the class Alpha-proteobacteria (Fig. S5).

### Immunological responses

**Figure 6:**
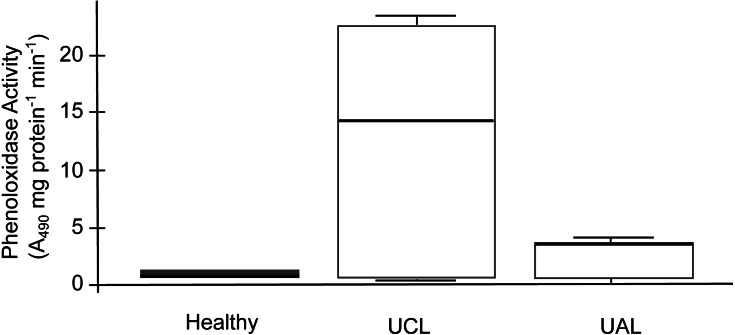
Phenoloxidase (PO) activity in mucus samples from healthy and unhealthy *Pseudodiploria strigosa* colonies. Box plot showing data from three independent experiments, expressed as A_490_ readings per mg of total protein per min. Mucus samples collected from healthy and unhealthy colonies, sampled close to the lesion (UCL) and away from the lesion (UAL).

The SML of healthy and unhealthy *P. strigosa* samples inhibited the growth of both tester strains (Table 2). Similarly, the MC of healthy *P. strigosa* samples inhibited the growth of *S. marcescens*; however, the growth inhibition of *Aurantimonas* sp. for the MC treatment was lower. The growth inhibition for *S. marcescens* by MC was also lower in the UCL and UAL samples compared with that caused by SML (Table 2). None of the treatments had significant differences among the condition of the colonies (*p* = 1 for *Aurantimonas* sp.; *p* = 1 for SML and *p* = 0.471 for MC, against *S. marcescens*). We did not find melanin in the tissues. We evidenced the lowest PO activity in mucus collected from healthy compared to unhealthy colonies. Conversely, the mucus of UCL samples showed the highest PO activity, while UAL samples showed intermediate levels of PO activity (Fig. 6). No significant differences were found among the three samples (*H* = 2, *df* = 2, *p* = 0.3679), probably due to the wide dispersion of the data for UCL samples and limited sample size. However, the data in Fig. 6 suggest that most of the UCL samples had 2 to 3 times higher PO activity than healthy and UAL samples.

**Table 2:** Antibacterial activity of the mucus from *Pseudodiploria strigosa* colonies against tester strains *Serratia marcescens* and *Aurantimonas* sp.

		**Surface Mucus Layer**^a^			**Mucus Complex**^b^		
		**H**	**UCL**	**UAL**	**H**	**UCL**	**UAL**
***Aurantimonas sp.***	**Frequency**	6/6	5/5	3/3	2/4	3/6	4/6
	**Proportion**	1	1	1	0.5	0.5	0.66
	Probability*			*p* = 1			*p* = 1
***Serratia marcescens***	**Frequency**	6/6	6/6	6/6	6/6	4/6	4/6
	**Proportion**	1	1	1	1	0.66	0.66
	Probability*			*p* = 1			*p* = 0.471

**Notes.**

a mucus layer without bacteria

b mucus layer without treatment

H healthy colonies UCL unhealthy colonies sampled close to lesion UAL unhealthy colonies sampled away from lesion

* probability calculated by Fisher Test among the condition of the sampled colonies, for each tester strain

## Discussion

We used the term white syndrome as an affliction that causes tissue loss exposing the skeleton, identified by generic signs with unknown etiology. In our study, lesions associated with WS correspond at the macroscopic level (Work & Aeby, 2006) to those described for WP-II, (Richardson et al., 1998b) and SCTLD, which may be a new coral disease in the region (NOAA, 2020). Common characteristics of WS-II and SCTLD are high lethality (Precht et al., 2016), multiple lesions in a single colony (NOAA, 2020), relatively fast lesion spread within a colony, and apparently density independent host relation (Precht et al., 2016). Unlike the disease in Florida that was coincident with a bleaching event in 2014 (Miller et al., 2016), in the Mexican Caribbean no major bleaching event was reported previous to the outbreak.

The WS outbreak on *P. strigosa* was characterized by low survival expectation of diseased colonies, coupled with an apparently constant disease incidence and no effective resistance to the disease progression once signs developed. Tissue loss rate of diseased *P. strigosa* varied, with a mean of 10.8 cm^2^ and s.d. of 7.8 cm^2^ that corresponds with ample ranges as reported for the WP-II (Richardson et al., 1998a; Richardson et al., 1998b). Many colonies remained in the diseased status for a long time before dying. This finding partially explains why after a peak in disease onset in early surveys (Fig. 1) there is not a subsequent death peak. An effect of this can also be observed in the last half of Fig. 1 showing the IR of death colonies increasing in winter, notwithstanding a marked decrease in the IR of diseased colonies. But the fate of diseased colonies was the same despite the speed of tissue loss, similar to other lethal coral diseases known in the Caribbean that progress over the colony at rates that overpass the growth rate of the coral host (Bruckner, 2016), eventually killing colonies that were hundreds of years old in a few months or years. The occurrence of multiple lesions and the high variability in lesion speed progression, partially explain why a significant correlation in colony size and time of death was not found. The emergence of lesions and their distribution within the remaining live tissue was apparentley random, suggesting that regardless of the healthy appearance of the remaining live tissue, the disease effect may be systemic over the whole colony, as is suspected for white-pox (Sutherland, Lipp & Porter, 2016) and suggested from our histological and microbial observations.

The decline in the IR from the peak in the first phase of the outbreak, when 76% of the colonies became diseased, to a third of the initial values, coincides with the winter season, when sea water temperatures decreased at the study site. However, loss of tissue on diseased colonies continued through the winter months, suggesting that lower sea water temperatures may not hinder lesion progression. Alternatively, as surface sea water temperatures at the study site in the 2018–2019 winter were relatively mild (range 24 to 27 °C; mean = 26 °C), temperature was not enough to hinder the lesion progression. The IR decline could also be associated with the reduction of the population size at risk of becoming diseased as a density dependent effect.

Currently there is no etiological characterization of this WS (Aeby et al., 2019) and the mechanisms by which a colony becomes diseased remain unknown. Histological observations demonstrate that the main host responses (necrosis, hypertrophy, and fragmentation) in *P. strigosa*, coincide with those observed for corals from the Central and Indo Pacific with similar gross lesions involving tissue loss (Work, Russell & Aeby, 2012; Sweet & Bythell, 2015). We did not find evidence of bacterial aggregates inside coral tissues that could be involved in the development of the disease. This is not uncommon in cases of white syndromes or any other tissue loss in corals, where pathogenic or commensal extracellular bacteria have not been observed affecting coral health (Aeby et al., 2016; Work, Aeby & Hughen, 2016). In colonies affected by WS in our study, we observed different organisms that were rarely in direct contact with the damaged tissue and those were not consistently present in all samples. White syndromes elsewhere have been reported as dynamic diseases with multiple possible causes including the action of some organisms such as ciliates, algae, or cyanobacteria (Work, Russell & Aeby, 2012). However, it is difficult to establish if the infections of eukaryotes arrived as primary or secondary invaders following a process of immunosuppression in the individual (Work, Russell & Aeby, 2012). In this study, only one case of necrosis was directly associated with ciliates. These organisms were recorded in nine different diseases (Sweet & Séré, 2016) and particularly, they have been reported in cases of WS in species from the Indo Pacific (Sweet & Bythell, 2015), Central Pacific (Work, Russell & Aeby, 2012), and the Caribbean (Croquer et al., 2016). Ciliates have been described as the agent involved in the pathogenesis of diseases due to its ability to consume the coral tissue in the lesion’s interface (Verde, Bastidas & Croquer, 2016), but also as secondary pathogens after initial bacterial infection (Sweet & Bythell, 2015). In any case, their presence at the margin of diseased tissues would make the repair of the epithelium more difficult (Work, Russell & Aeby, 2012). Finally, the histological alterations in the polyps were seen from the basal (BBW) to the surface body wall (SBW), revealing a bottom-up pattern in the deterioration process, suggesting that the pathogenesis of this WS might begin before macroscopic signs are evident in the colony. This finding is quite unexpected for healthy colonies, and even for healthy tissues of colonies affected by WS, where tissues remained unaltered without evidence of necrosis, fragmentation, or atrophy (Sweet & Bythell, 2015; Pollock et al., 2017). Considering that gross lesions are the main manifestation of diseases in epidemiological studies on reef corals, this could lead to an underestimation of the prevalence of white syndromes.

The absence of bacterial aggregates in direct association with damaged tissue, is not indicative *per se* that microorganisms do not play a role in the disease. The bacterial community inhabiting the SML of healthy *P. strigosa* colonies, showed abundant representatives of classes Gamma-, Alpha-proteobacteria, and Bacteroidia, usually found associated with *P. strigosa* from other Caribbean localities (Meyer et al., 2016) and other coral species (Huggett & Apprill, 2019; Meyer et al., 2019). In contrast, different members of Gamma-proteobacteria, Clostridia, and Bacilli, were abundant in unhealthy colonies. In the class Bacteroidia, six genera were found with significant changes, including the genus *Roseimarinus*, the most abundant taxon we detected in UCL colonies (39.27 ± 23.37%). This genus has been reported in flocculated sediments associated with shrimp farms, recognized as part of a biomarker bacterial group for marine sediments with high organic matter load (Verhoeven et al., 2018). However, the identity of this genus will need further validation, as the closest relative known in the RefSeq database for the most abundant *Roseimarinus* ASV was of a different genus to the one assigned by SILVA. We also detected relatively abundant Campylobacterales (mostly in the genus *Acrobacter*) in unhealthy colonies, in agreement with findings in colonies affected with SCTLD in Florida (Meyer et al., 2019). A more recent study on SCTLD in Florida reported a higher abundance of orders Rhodobacterales and Rhizobiales in the lesion of the coral species *Stephanocoenia intersepta, Diploria labyrinthiformis, Dichocoenia stokesii*, and *Meandrina meandrites,* and in sediment samples, suggesting sediments may play a role in the disease (Rosales et al., 2020). Rhodobacterales was also identified as significantly increased in abundance in Acroporid corals affected with a white syndrome (Pollock et al., 2017) and in WP-II affected colonies of *Orbicella* (*Montastrea*) *faveolata* (Sunagawa et al., 2009). Although both orders were also identified in our samples, we did not find similar trends: Rhodobacterales being more abundant in healthy and UAL colonies. However, Rhodobacterales, Rhizobiales, and other taxa like Vibrionales, Flavobacteriales, and Alteromonadales, have been reported as typically increasing in abundance in response to stress (McDevitt-Irwin et al., 2017). Overall, these studies suggest that opportunistic, abundant bacteria differ in similar afflictions (Mera & Bourne, 2018).

The species richness and diversity of the bacterial community associated with the mucus layer varied within and among the condition of the colonies with no discernible patterns. However, a differential abundance analysis (DESeq2) was more informative, showing 16 ASVs shifting significantly in unhealthy colonies (UCL) that suggest destabilization of this community (Zaneveld, McMinds & Vega-Thurber, 2017). Unhealthy colonies consistently showed an opportunistic bacterial overload. However, it is not clear if such growth was being supported by decaying tissues or was contributing to the deterioration process. Changes in the bacterial community of coral holobionts in response to environmental stress, may affect the defense mechanisms contributing to the sensitivity of the host to bleaching and disease (Bourne, Morrow & Webster, 2016). According to our observations, the antibacterial activity of the mucus layer effectively inhibited the growth of two potential pathogens of corals, regardless of the health status of the coral or the location into the colony (close or away from the lesion), as has been reported for this coral species with black band disease (Rivera-Ortega & Thomé, 2018) and also for other diseased corals (Palmer, Bythell & Willis, 2010; Ocampo et al., 2015). Phenoloxidase activity in *P. strigosa* behaved similarly to that reported in colonies affected by black band disease (Rivera-Ortega & Thomé, 2018), although the overall values measured for this activity in the mucus of healthy and WS affected colonies (close and away to the lesion) were several orders of magnitude higher. These results suggest that the immunological response in *P. strigosa* was enhanced as reported in compromised, infected, and thermally stressed tissues of many coral species (Palmer & Traylor-Knowles, 2012). Our histological observations did not confirm an infectious process in WS affected colonies, suggesting that the immunological response that we measured could have been intended to fight a bacterial overgrowth or a destabilization of the bacterial community (Bourne, Morrow & Webster, 2016; Glasl, Herndl & Frade, 2016). Moreover, we did not find melanin deposits into the coral tissue in healthy or unhealthy colonies, as has been previously reported in apparently healthy *P. strigosa* (Mydlarz & Palmer, 2011). A transcriptional study of immune-related transcripts in *P. strigosa* showed a high complexity of its immune mechanisms, suggesting a resilience of this species to coral diseases (Ocampo et al., 2015). Notwithstanding, *P. strigosa* together with other meandroid corals, was one of the least resistant species at our study site.

## Conclusions

The complexity of host-microbial interactions makes the understanding of WS in *Pseudodiploria strigosa* problematic, in particular when the information acquired from microbial, immunological, and histological data do not suggest specific causes, but a sudden deterioration of the tissues that negatively impacted the survival of colonies. The main findings of our study support the view that no specific pathogens seem to be involved in the WS in *P. strigosa*, although an active immune response within diseased colonies suggests disruptive factors involved in degrading the coral’s tissues that allow opportunistic bacteria to grow. Finding different opportunistic bacteria and other pathogens associated with a so-named disease is no proof of causality (Hill, 1965), and the possibility that in one setting a presumed pathogen is active and in another is not, has been demonstrated repeatedly (Pirofski & Casadevall, 2012). This opens the possibility that deterioration patterns of individual colony’s health may be caused by environmental assaults, severely diminishing the ability of corals to regulate their microbiome, influencing coral health. Whatever the cause of degrading tissues in this WS, perhaps experimental tools can contribute to disentangle the etiology of this disease (Work & Metemeyer, 2014; Rosales et al., 2019).

In the Caribbean region coral reefs live under elevated sea surface temperature conditions (Muñiz-Castillo et al., 2019) and poor reef-water quality (Harvell et al., 2007; Cooper, Gilmour & Fabricius, 2009). At our study site, corals including *P. strigosa* may also be affected by brown tides from the decomposition of beached *Sargassum* (Van Tussenbroek et al., 2017; Rodríguez-Martínez et al., 2019), further introducing nutrients and organic material into the water. By the end of our study (306 days) only 8 colonies remained in an apparently healthy condition and later, informal observations, failed to find five of those colonies alive. Longer lasting colonies with an apparently healthy status may survive, for a time, due to a combination of genotype resistance (Libro & Vollmer, 2016), a disease resistant microbiome (Rosales et al., 2019), and low susceptibility to environmental heat stress (Schoepf et al., 2019). However, continuing and increasing environmental stress affects coral metabolic performance which in turn implies reduced growth, reproductive output, ecological competitiveness (Hughes & Tanner, 2000), and compromised immunity (Harvell et al., 2007). If environmental deterioration is the primary cause of the continuing emergence and re-emergence of coral lethal diseases, we consider that resistant colonies to one disease would eventually be susceptible to another. In this scenario, the only true option to effectively help preserve the coral reef biodiversity and coral reef services, is to restore the environmental quality of reef waters at the local scale and reduce greenhouse gases at the global scale.

##  Supplemental Information

10.7717/peerj.10695/supp-1 Supplemental Information 1 Supplementary reference to QIIME 2 pipeline

10.7717/peerj.10695/supp-2 Supplemental Information 2 Tables S1, S3, and S4 and Figures S1–S7

10.7717/peerj.10695/supp-3 Table S2 Relative abundance of all ASVs, raw data (after rarefaction)

10.7717/peerj.10695/supp-4 Supplemental Information 4 Raw data for field surveys

10.7717/peerj.10695/supp-5 Supplemental Information 5 PO raw data Protein and A_490_ readings for PO activity is sampled mucus from healthy and unhealthy colonies.

## Additional Information and Declarations

### Field Study Permissions

The following information was supplied relating to field study approvals (i.e., approving body and any reference numbers): Collection of samples was done under permit number PPF/DGOPA-033/19 from CONAPESCA.

### Data Availability

The following information was supplied regarding data availability: Sequences raw data are available at NCBI: PRJNA610838. Raw data from PO activity, the condition of colonies, and field surveys are available in the Supplemental Files.
