# Supplementary material for: Local dynamics of a white syndrome outbreak and changes in the microbial community associated with colonies of the scleractinian brain coral Pseudodiploria strigosa
Source: PeerJ. 2021 Feb 2;9:e10695. doi: 10.7717/peerj.10695 (PMC7863780; doi:10.7717/peerj.10695)
Supplement: Supplemental Information 2 [file peerj-09-10695-s002.pdf]

**Supplementary Tables and Figures - Thomé et al.**

**Table S1. Size and percentage of damaged tissue for sampled *Pseudodiploria strigosa* colonies.** Healthy colonies did not have visual signs of disease. For unhealthy colonies, UCL refers to samples taken close to the lesion; UAL refers to samples taken away from the lesion. Colonies successfully sequenced indicated with an asterisk.

| **Healthy colonies** | Diameter (mm) | **Unhealthy colonies** | Diameter (mm) | Damaged tissue (%) |
|---|---|---|---|---|
| **H1*** | 254 | **UCL1/UAL1*** | 255 | 23.49 |
| **H2*** | 255 | **UCL2/UAL2*** | 255 | 49.84 |
| **H3*** | 227 | **U3CL*** | 249 | 55.92 |
| **H4** | 255 | **U4** | 245 | 66.54 |
| **H5** | 250 | **U5** | 253 | 27.81 |
| **H6** | 228 | **U6** | 254 | 77.18 |

**Table S2.** Relative abundance of all ASVs, raw data (Excel file, after rarefaction). Uploaded separately.

**Table S3.** Alpha diversity indexes calculated for all colonies sampled.

| Colony | Obs. | Chao1 | ACE | Shannon | Simpson |
|---|---|---|---|---|---|
| **H1** | 202 | 203 | 202.29 | 3.57 | 0.91 |
| **H2** | 147 | 147.5 | 147.63 | 3.06 | 0.85 |
| **H3** | 111 | 111.25 | 111.43 | 2.14 | 0.72 |
| **UAL1** | 155 | 155 | 155 | 3.11 | 0.89 |
| **UAL2** | 131 | 131.2 | 131.57 | 2.83 | 0.85 |
| **UCL1** | 233 | 233 | 233.21 | 4.15 | 0.97 |
| **UCL2** | 115 | 115 | 115 | 2.51 | 0.74 |
| **UCL3** | 112 | 117.14 | 115.61 | 2.32 | 0.70 |

H1-3, healthy colonies; UAL1-2, unhealthy colonies sampled away from lesions; UCL1-3, unhealthy colonies sampled close to lesions.

**Table S4**. Relative abundance (%) in *Pseudodiploria strigosa* colonies and water samples, for two bacterial Orders implicated in WS and SCTLD.[1]

| | **H1** | **H2** | **H3** | **UAL1** | **UAL2** | **UCL1** | **UCL2** | **UCL3** | **WH** | **WD** |
|---|---|---|---|---|---|---|---|---|---|---|
| **Rhodobacterales** | 5.90 | 2.55 | 11.61 | 7.83 | 10.67 | 2.11 | 0.48 | 0.19 | 11.56 | 17.20 |
| **Rhizobiales** | 0.05 | 0.12 | 0.14 | 0 | 0.04 | 0.49 | 0 | 0.02 | 0 | 0.14 |

[1] According to Pollock et al, 2017; Rosales et al., 2020
All data are for single samples.

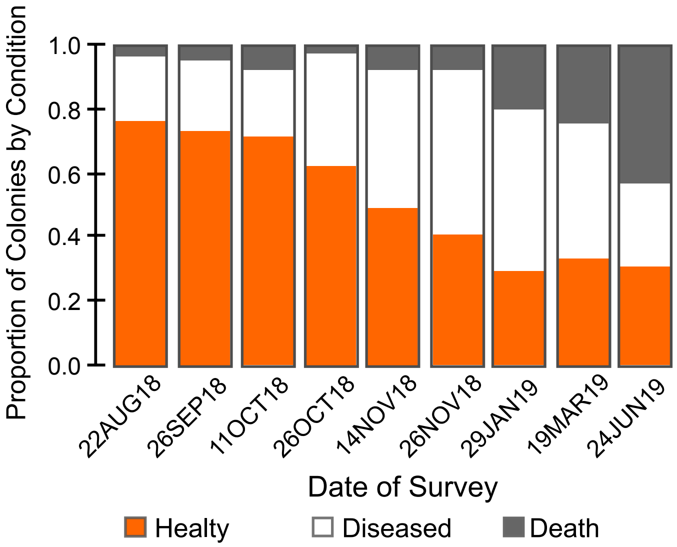


**Figure S1. Status of *Pseudodiploria strigosa* colonies.** Colonies sampled on different surveys conducted in a rear reef site near Puerto Morelos in the NE Mexican Caribbean. Colonies (95 total) were mapped and followed within an area of 435 m$^2$ throughout 306 days. The proportion of healthy, diseased, and dead colonies is given for 100 colonies per survey day.

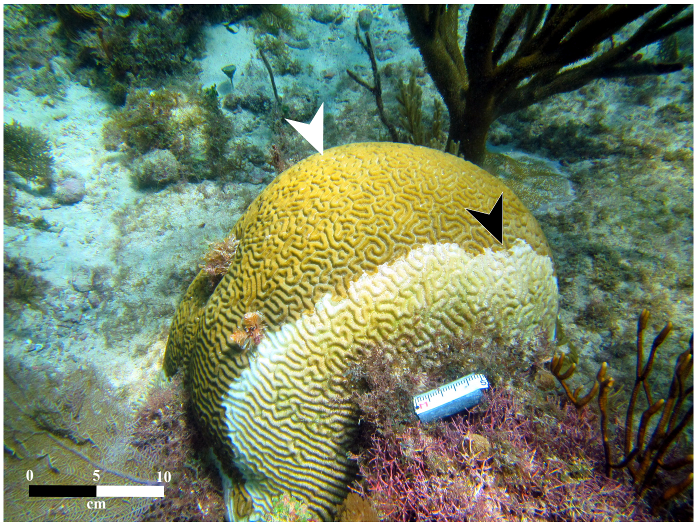


**Figure S2. *Pseudodiploria strigosa* tissue sampling.** The photograph illustrates the location of sampling points for unhealthy colonies, at ≤ 1 cm from the lesion border (black arrowhead, UCL samples) and 15 cm away from the lesion border, over healthy-looking tissue (white arrowhead, UAL samples). Photo credit: Eric Jordán-Dahlgren.

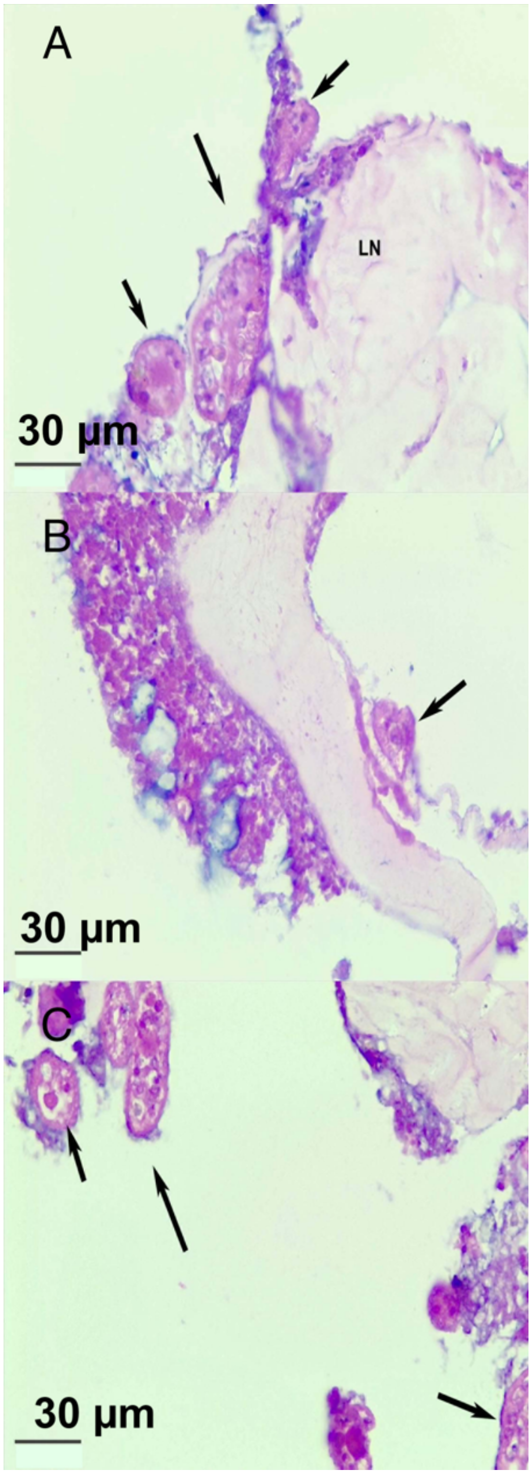


**Figure S3. Microphotographs of ciliates in the proximity of tissue affected (UAL) by WS in *Pseudodiploria strigosa* colonies.** (**A**) Ciliates (black arrows) in direct contact with necrotic tissue (LN). (**B**) Ciliate (black) arrow next to the mesoglea within the mesentery of the individual. (**C**) Several ciliates (black arrows) next to necrotic tissue (LN) within the mesentery.

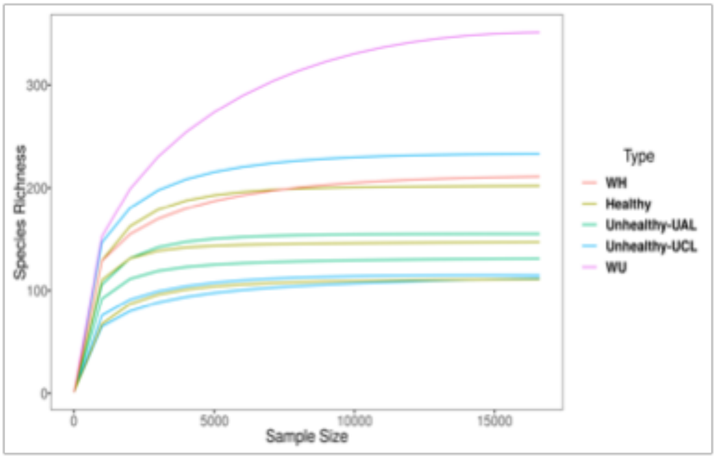


**Figure S4. Accumulation curves for ASVs resolved per sample.** Total number of reads was 261,176 normalized to 16,600 per sample after rarefaction.

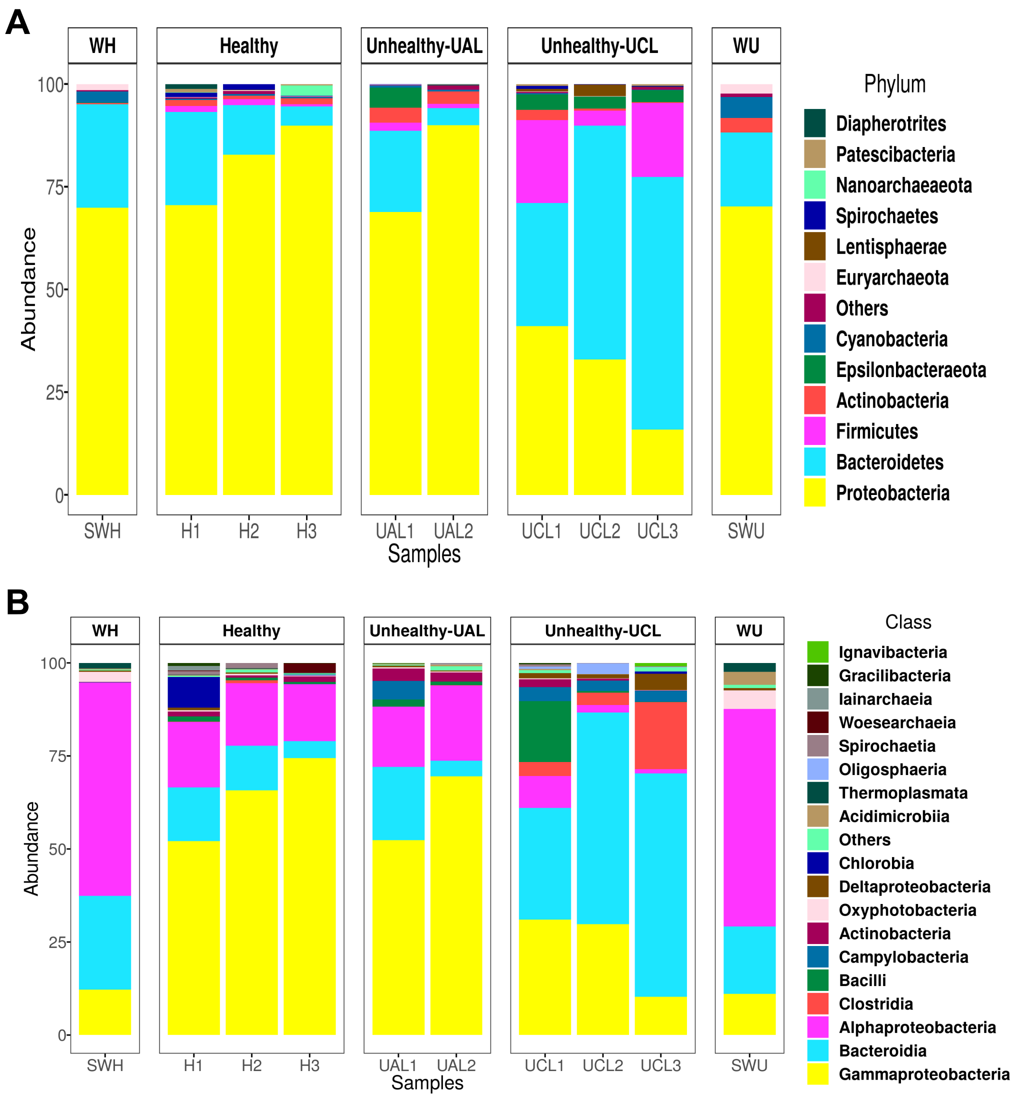


**Figure S5. Bacterial community associated with the surface mucus layer of *Pseudodiploria strigosa* colonies.** Relative abundance of assigned (**A**) Phylum and (**B**) Class to ASVs from sampled colonies. Sample identity as follows: Healthy colonies: H1-3, unhealthy colonies sampled away from lesion: UAL1-2, unhealthy close to lesion: UCL1-3, seawater sample close to a healthy colony: WH, seawater sample close to an unhealthy colony: WU. Phyla with < 1% of abundances were grouped in the "Others" category.

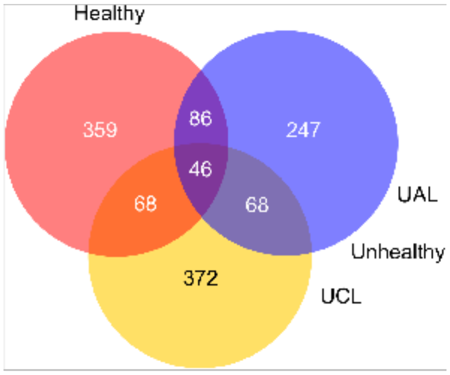


**Figure S6. Venn diagram showing total number of ASVs for the colonies sampled.** The numbers in the center of each colored circle refer to the total number of ASVs for each category; numbers on intersections show shared ASVs between and among categories. Note that the sample size was different for UAL with only 2 samples, while Healthy and UCL conditions had 3 samples each.

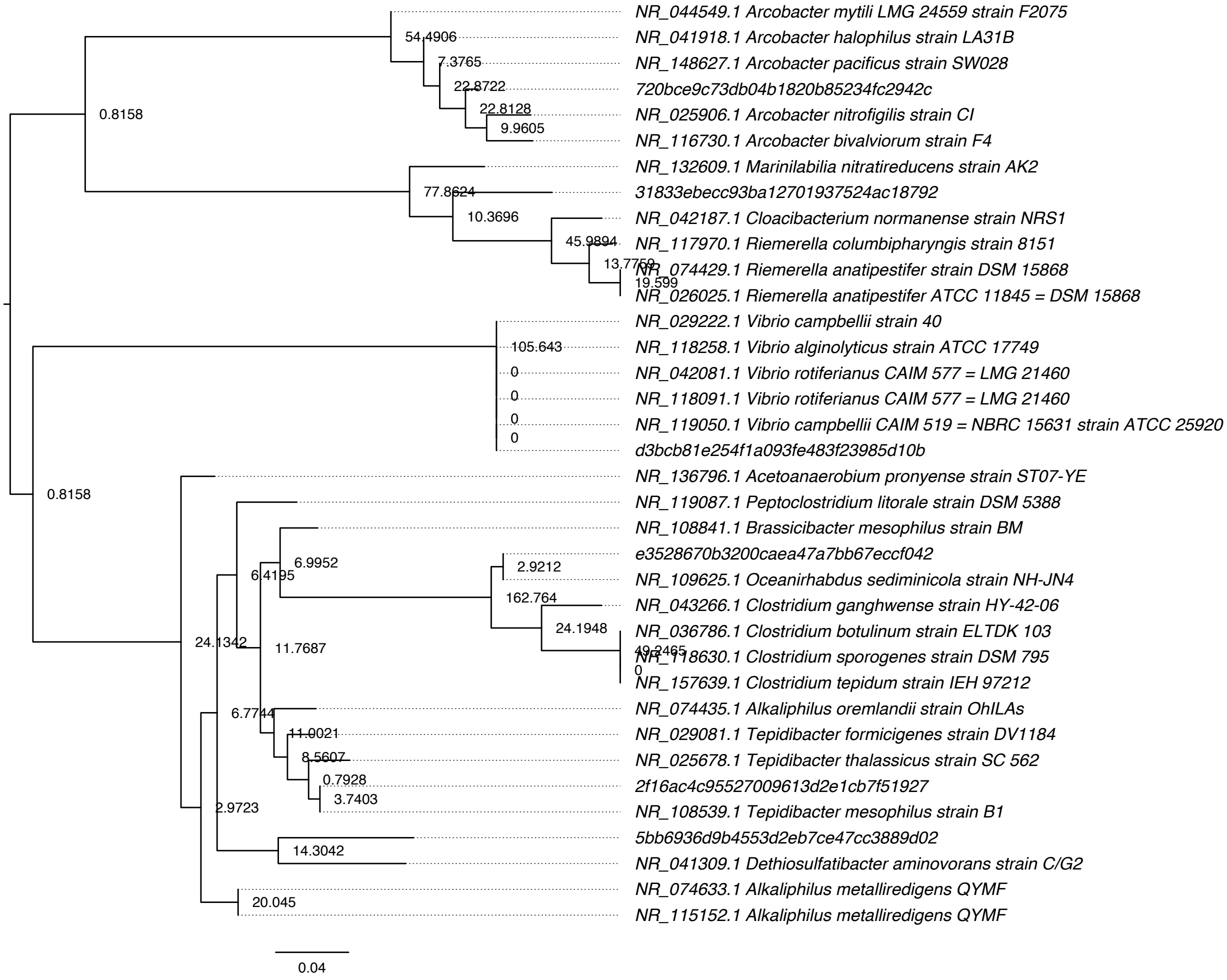


**Figure S7. Phylogeny analysis for the most abundant ASVs within the six genera identified in unhealthy colonies.** This analysis was done using as reference the NCBI RefSeq database.
